# Clinical time course of the neglected giant sebaceous gland carcinoma

**DOI:** 10.22336/rjo.2021.59

**Published:** 2021

**Authors:** Mehmet Serhat Mangan, Zuhal Ozcan, Nilgun Ozkan Aksoy

**Affiliations:** * Haydarpasa Numune Education and Research Hospital, Sadik Eratik Eye Institute, Istanbul, Turkey; ** Department of Pathology, Haydarpasa Numune Education and Research Hospital, Istanbul, Turkey; *** Department of Ophthalmology, Sakarya University Education and Research Hospital, Sakarya, Turkey

**Keywords:** sebaceous gland carcinoma, eyelid, clinical time course, orbital exenteration

## Abstract

In this report, we discussed the progression from the initial presentation until surgical intervention, clinical course, and devastating outcome of a neglected giant sebaceous gland carcinoma of the eyelid in a poorly compliant elderly patient.

A 79-year-old woman was referred for treatment of a giant ulcero-nodular lesion in the right upper eyelid. Nine months before, an orange lesion arising from the tarsal conjunctiva in the upper eyelid was observed in her examination undergone in the healthcare center where she initially presented, and the cornea appeared transparent. Surgical excision was recommended, which she declined. The examination three months before in the same center revealed that the lesion invaded the globe and anterior segment architecture could not be visualized. She was then recommended surgical removal of the eyeball, which she also refused. Radiological imaging demonstrated a 33x35 mm mass lesion in the superior lateral of orbit with exophytic growth and invasion of the globe and no systemic metastases were found. Total orbital exenteration surgery was immediately planned and performed. Histopathological examination revealed sebaceous gland carcinoma.

Elderly patients with poor compliance should discuss their condition with a psychiatrist and should be managed by a multidisciplinary approach. This way, patients with eyelid malignancies can be encouraged to undergo surgery and receive early treatment, decreasing the need for exenteration, improving clinical outcomes, and reducing the risk of morbidity and mortality.

## Introduction

Sebaceous gland carcinoma is a rare malignant tumor of the eyelid, comprising 1% to 5.5% of all eyelid tumors [**1**-**4**]. Postponing and neglecting treatment, especially in the elderly patients with low compliance, can lead to poor clinical outcomes [**5**-**9**]. To the best of the authors’ knowledge, there is no study in literature that presents the progression of a sebaceous gland carcinoma with clinical photographs. 

In this case report, we discussed the progression from the initial presentation until surgical intervention, clinical course, and devastating outcome of a neglected giant sebaceous gland carcinoma of the eyelid in a poorly compliant elderly patient.

## Case report

Written informed consent was obtained from the patient for the publication of this case report and any accompanying images. This case report follows the ethical principles outlined in the Declaration of Helsinki. 

**Fig. 1:**
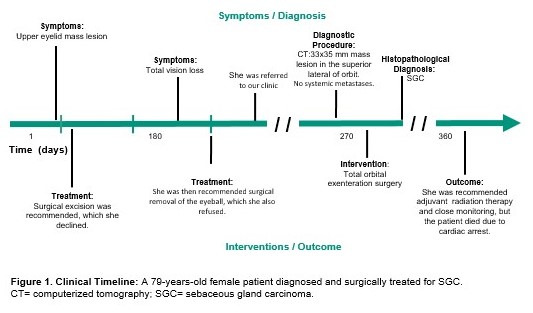
Clinical Timeline: A 79-year-old female patient diagnosed and surgically treated for SGC. CT = computerized tomography, SGC = sebaceous gland carcinoma

A 79-year-old female patient was referred to our clinic for orbital exenteration surgery (**Fig. 1**). 

**Fig. 2:**
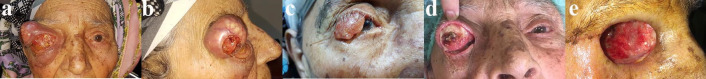
Patient’s clinical time-course **(a)** Frontal view of the patient at the last examination before surgery. **(b)** Temporal view of the patient at the last examination before surgery. **(c)** Photo of the patient 9 months before at the initial presentation. **(d)** Photo of the patient 3 months before at the initial center. **(e)** Photo of the patient after exenteration surgery

Her ophthalmological examination revealed a giant ulcero-nodular mass lesion in the right upper eyelid extending to superior temporal. Anterior segment details could not be visualized and there was no light perception (**Fig. 2a, b**). Nine months before, a yellowish orange mass arising from the tarsal conjunctiva in the upper eyelid was observed in her examination undergone in the healthcare center where she initially presented, and the cornea appeared transparent (**Fig. 2c**). Surgical excision was recommended, which she declined. Examination three months before in the same center revealed that the mass lesions invaded the eye globe and anterior segment architecture could not be visualized (**Fig. 2d**). She was then recommended surgical removal of the eyeball, which she also refused. 

**Fig. 3:**
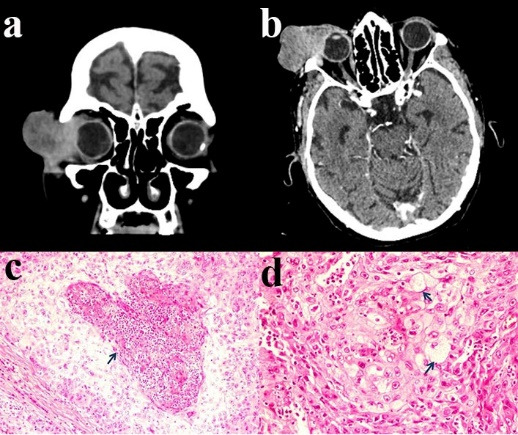
**(a)** Coronal images of patient’s computerized tomography study show a 33x35 mm mass lesion in the superior lateral of right orbit with exophytic growth. **(b)** Axial images of patient’s computerized tomography study show the mass invading the eye globe. **(c)** Nested pattern tumor cells with central necrosis (arrow) in histopathological examination. **(d)** Cells with foamy, vacuolated cytoplasm and distinct cell borders (arrow) in histopathological examination

Radiological imaging studies showed a 33x35 mm mass lesion in the superior lateral of right orbit with exophytic growth and invasion of the eye globe (**Fig. 3a, b**) and no systemic metastases were found. Total orbital exenteration surgery was planned. Because the tumor invaded the entire upper eyelid, eyelid preserving surgery could not be executed. Resection was performed with a 5-mm surgical margin and the skin margin was assessed with rapid frozen examination. After total orbital exenteration, the surgical site was left to heal by secondary intention. Histopathological examination of the excised material revealed nested pattern tumor cells with central necrosis (**Fig. 3c**) and cells with foamy, vacuolated cytoplasm and distinct cell borders (**Fig. 3d**), which was consistent with classic subtype sebaceous gland carcinoma. There were no signs of fistula or infection in the 2nd postoperative month follow-up (**Fig. 2e**). She was recommended adjuvant radiation therapy and close monitoring, but unfortunately the patient died due to cardiac arrest.

## Discussion

Unlike other malignancies of the body, eyelid malignancies may result in loss of vision when not treated early, posing one of the greatest challenges that oculoplastic surgeons encounter [**5**-**8**]. In this case report, the patient had a periocular sebaceous gland carcinoma with invasion of the right orbit and eye globe at the time of referral to our center and unfortunately completely lost sight. However, at the time of initial presentation, the eyelid mass was localized and small, and her visual acuity was preserved. Sebaceous gland carcinoma is more frequent in women and is usually diagnosed in the 6th to 7th decade of life, as in our case. It typically presents in the upper eyelid [**1**-**4**]. Sebaceous gland carcinoma usually arises from the meibomian glands and its differential diagnosis is especially important as it can mimic basal cell carcinoma, squamous cell carcinoma, and malignant melanoma. Surgical excision is the primary treatment modality for sebaceous gland carcinoma, while chemotherapy and radiation therapy can be employed as adjuvant treatment [**1**,**2**]. Therefore, surgery should be executed immediately after diagnosis. 

Declining surgical treatment can cause irreversible damage, especially in elderly patients with poor compliance [**5**-**10**]. Increased risk of mental diseases, including dementia and schizophrenia with advanced age, can diminish the patient’s compliance [**5**,**6**,**8**,**9**]. There are few cases with basal cell or squamous cell carcinoma who refused surgical treatment in the literature [**5**,**6**,**8**,**9**]. Obtaining an informed consent from poorly compliant elderly patients who ignore surgical treatment options is a challenging issue with a lack of consensus on its management. 

## Conclusion

Elderly patients with poor compliance should have a thorough discussion with a psychiatrist and should be managed with a multidisciplinary approach. This way, patients with eyelid malignancies can be encouraged to undergo surgery and receive early treatment, decreasing the need for exenteration, improving clinical outcomes, and reducing the risk of morbidity and mortality.

**Conflict of Interest statement**

Authors state no conflict of interest.

**Informed Consent and Human and Animal Rights statement**

Informed consent has been obtained from all individuals included in this study.

**Authorization for the use of human subjects**

Ethical approval: The research related to human use complies with all the relevant national regulations, institutional policies, is in accordance with the tenets of the Helsinki Declaration, and has been approved by the review board of Haydarpasa Numune Education and Research Hospital, Sadik Eratik Eye Institute, Istanbul, Turkey.

**Acknowledgements**

None.

**Sources of Funding**

None.

**Disclosures**

None.
